# Rice Husk-Based Adsorbents for Removal of Metals from Aqueous Solutions

**DOI:** 10.3390/ma16237353

**Published:** 2023-11-26

**Authors:** Svetlana Yefremova, Askhat Kablanbekov, Baimakhan Satbaev, Abdurassul Zharmenov

**Affiliations:** 1 National Center on Complex Processing of Mineral Raw Materials of the Republic of Kazakhstan RSE, Almaty 050036, Kazakhstan; kablanbekov_as@mail.ru (A.K.); jarmen56@mail.ru (A.Z.); 2 School of Materials Science and Green Technologies, Kazakh-British Technical University, Almaty 050000, Kazakhstan; 3 Astana Branch of the National Center on Complex Processing of Mineral Raw Materials of the Republic of Kazakhstan RSE, Astana 010000, Kazakhstan; fnc-astana@mail.ru

**Keywords:** rice husk, biosorbent, adsorbent, adsorption, water purification, heavy metals’ uptake, radionuclides’ removal, precious, rare and rare-earth metals’ concentration and preconcentration

## Abstract

Adsorption is one of the main methods of water purification. Novel advanced, eco-friendly, cost-effective adsorbents with high adsorption capacity and selectivity are required to remove pollutants from aqueous solutions. Plant polymers are viewed as both prospective adsorbents and as raw materials to produce them instead of conventional adsorption materials. There is widespread interest in using rice husk as a universal sorbent to remove different contaminants from aqueous media because of its surplus availability, low cost, and high content of oxygen containing functional and silanol groups as active sites for adsorptive extraction. Different methods of heat and chemical treatments have been developed to improve the sorption properties of raw rice husk. Unmodified rice husk and rice-husk-based sorbents have been tested to uptake non-ferrous, ferrous, minor, precious, rare, and rare-earth metals and radionuclides from artificial and industrial solutions, natural contaminated water, and industrial wastewater. This review summarizes the results of numerous studies and characterizes the current state of work in this area, with recommendations for further development.

## 1. Introduction

Population growth, scientific and technological progress, industrialization, and agricultural development are associated with an increased burden on the environment. The huge amounts of domestic, industrial, and agricultural waste pose a global problem. In this regard, waste processing has been an urgent issue for more than a decade. Despite that there are quite effective examples of the use of various wastes in different fields [1]—for example, metallurgical waste in the cement industry [2,3], or together with the use of polymers, including the replacement of expensive ethylene-vinyl acetate with waste from the production of cotton oil gossypol resin—in the production of protective coatings [4], the problem, obviously, due to its scale, is far from being solved. 

The scientific community is concerned about the state of water resources, the quality of drinking water, and water for everyday use, which are polluted due to the discharge of a large amount of wastewater containing compounds harmful to human health, flora, and fauna [5,6]. Among the numerous methods used for wastewater treatment, adsorption is considered the easiest to perform and the most efficient in terms of the environment and economics [5,7]. Active carbons, polymer materials, natural minerals, and zeolites are traditionally used as adsorbents [8,9]. However, on the one hand, the growing demand for adsorption materials, and on the other hand, the increasing requirements for their quality (high sorption efficiency, biodegradability, and selectivity), make it necessary to search for alternative sources for their production [10,11]. In this regard, scientific research has intensified on the use of different types of waste in wastewater treatment plants or waste-based adsorbents’ production to treat natural objects and industrial wastewater [12]. The creation and application of biosorbents has matured as an independent scientific direction [13]. Moreover, these biosorbents are offered for use not only for the purposes of cleaning water systems from various pollutants, but also for the extraction and preconcentration of precious, rare, and rare-earth metals [14,15]. It is believed that natural biopolymers, due to the presence of ether bond (−C−O−C−) and hydroxyl (−OH) groups, are able to form complexes with metal cations, which allows them to be considered as potential adsorbents [16].

Rice husk, which is a multi-tonnage waste generated during rice production, has firmly taken its place among the biopolymer raw materials required to create adsorbents [17]. The main components of rice husk are cellulose, lignin, hemicellulose, and amorphous silicon dioxide [18], which determine the good sorption properties [19,20,21,22,23,24,25]. Chuah et al. [26] in their review discussed the sorption of heavy metals and dyes using rice husk and rice-husk-based active carbons. It follows from the reviews in [26,27] that previous studies were not systematic and did not find wide practical application. Moreover, it was noted that there are certain difficulties in the process of activating rice-husk-based carbons [26]. In this regard, it was of interest to review the available literature over the past 15 years to assess progress in this area. The analysis showed that research in this direction is not only continuing but also expanding, especially in terms of modifying rice husk to improve its sorption characteristics. 

This review includes an analysis of studies on sorption of non-ferrous, ferrous, minor, radioactive, precious, rare, and rare-earth metals by unmodified and modified rice husk. For each sorbent, the methodology for its preparation is presented, with appropriate references to the original sources where the details of the experiment can be found. The sorption efficiency of new materials toward different types of metals is analyzed depending on the sorption process conditions (initial adsorbent concentration, solution pH, sorbent dose, and sorbent–solution contact time) in each specific case. Information on the kinetics, mechanisms, and thermodynamic parameters of metal ion adsorption is presented. An analysis of the rice-husk-based adsorbents’ effectiveness depending on their production methods is carried out. A general characterization of the background and the current state of the studied issue is presented, and directions for further development of research on the sorption of metals from aqueous medium by rice-husk-based sorbents are proposed.

## 2. Rice-Husk-Based Adsorbents’ Production

Various methods of production of adsorbents using rice husk (RH) have been offered for the removal of different kinds of metals. Using unmodified plant raw material has been examined as well. Since even unmodified RH-based sorbents differ from each other in their preparation methods, a list of RH-based adsorbents reviewed in this work and a brief description of their production methods is presented in Table 1. 

## 3. Adsorption of Non-Ferrous Metals

### 3.1. Adsorption of Lead

Lead is considered one of the most toxic pollutants in the environment. In this context, many scientists are working to find low-cost sorbents for the removal of Pb^2+^ ions from water (Tables S1 and S2). Singha et al. [28] and Gupta S. et al. [29] used rice husk only washed with water under different conditions (Table 1). Gupta N. et al. [30] and Aluyor et al. [31] produced a hybrid carbon-silica gel adsorbent and hydrogen peroxide-modified rice husk for treating plant waste with NaOH solutions in the presence of ethylene glycol and H_2_O_2_ after alkali treatment, respectively. Masoumi et al. [32] prepared a crosslinked nanoparticle sorbent using C_4_H_6_O_6_-modified rice husk and a copolymer. Fan et al. [33] carried out carbonization of vegetable raw material with sulfuric acid, followed by ammonium persulfate oxidation, to prepare a high-performance sorbent for the removal of Pb (II) from solutions. Ullah et al. [34] carbonized rice husk soaked in a 1.0 M HNO_3_ solution for 24 h at 400–800 °C in an air-free atmosphere. Zharmenov et al. [35] heated rice husk in a rotary furnace at 400 °C under an off-gas atmosphere for 30 min, cooled it without air to room temperature, and activated the carbonized product using water vapor at 850 °C for 30 min; then, the cooled activated product was subsequently treated with a 70 g·dm^−3^ NaOH solution at 70–80 °C for 2 h.

Roha et al. [36] studied Pb^2+^ sorption by various kinds of rice-husk-based sorbents (RH1, RHB, EDTA-RHB, and MB; Table 1 and Table S2) in comparison with other metal cations (Cu^2+^ and Cd^2+^) from a mixed solution of these potentially toxic elements (PTEs). It was shown that MB was the best sorbent in relation to all PTEs, although the percentage of lead removal was the lowest (Tables S2–S4). Xu et al. [37] studied lead removal as well as other PTEs from mono- and multi-metal systems containing Pb, Cu, Zn, and/or Cd and observed an opposite trend. The load of metals removed by RHBC from both the mono- and multi-metal solutions was the highest in the case of Pb^2+^ ions (Tables S1–S8). This was due to the highest ionic potential of lead ions (0.22), providing the strongest complexation of Pb^2+^ with oxygen in RHBC phenolic-OH [37]. 

The highest affinity of different sorbents produced using rice husk as a feedstock to Pb^2+^ (Tables S1 and S2), in comparison to Cd^2+^, Zn^2+^, Cu^2+^, Ni^2+^, Hg^2+^, Co^2+^, Mn^2+^, Cr^3+^, and Fe^3+^ (Tables S3–S5, S7–S9, and S10–S13), was presented by Akhtar et al. [38], Krishnani et al. [39], and Sheveleva et al. [40]. Moreover, the materials treated with alkali showed the highest sorption properties in comparison to rice husk modified with acids. Although C–O–Si bonds were present in rice husk after alkali treatment, silicon removal was achieved almost completely [71]. It can be suggested that as a result, new active sites form. An added heat treatment as well as independent heat treatment at low temperatures enhances this process due to thermal destruction and removal of volatile compounds. As a result, these sorbents removed Pb (II) well, not only from single-component solutions but also from complex systems. For example, RH_wNcT_ exhibited 98% removal of lead from industrial wastewater spiked with 10 mg·dm^−3^ of Pb solution [38] (Table S1). RH_NaOH_ sorbent provided separation of lead and copper from cadmium in the Pb^2+^-Cu^2+^-Cd^2+^ solution (Tables S2–S4). In contrast, lead removal from the multi-component solution containing Cr (III), Fe (II), Mn (II), Ni (II), Zn (II), and Pb (II) using another sorbent (RHC_a_-CO_2_) in column experiments was the lowest [41], as shown above for batch experiments. 

Based on the research papers cited in [29,30,31,32,33,34,37,38,39], it was found that the Langmuir model fit well with the experimental data obtained on RH_tw_, RH_g_, RH_HP_, RH_TA_, RH_C-OX_, RH_C-Si_-400, RH_C-Si_-600, RHBC, RH_wNcT_, and RH_a_. Conversely, only RH_b_, RH_C-Si_-800 °C, MB, and SCActA presented heterogenic adsorption, wherein the Freundlich equation was applied [28,34,35,36]. The adsorption kinetics of Pb (II) are determined by the pseudo-second-order kinetic model for RH_C-OX_, RH_C-Si_-400, RH_C-Si_-600, RH_C-Si_-800, RH_TA_, and RH_tw_ sorbents [29,32,33,34], and the intra-particle diffusion model for RH_b_ sorbent [28]. Both the pseudo-second-order and pseudo-first-order kinetic models are applied for MB [36]. The adsorption onto RH_wNcT_ demonstrates first-order rate kinetics and a partial intra-particle diffusion mechanism [38]. According to the thermodynamic parameters presented in [28,30,33,38], Pb(II) sorption processes on rice-husk-based sorbents were spontaneous (negative ΔG° values were calculated in the case of RH_b_, RH_g_, RH_C-OX_, and RH_wNcT_) and predominantly random (positive ΔS° values were calculated in the case of RH_b_, RH_g_, and RH_C-OX_), and endothermic (positive ΔH° values were calculated in the case of RH_b_ and RH_C-OX_) or exothermic (negative ΔH° values were calculated in the case of RH_g_ and RH_wNcT_) in nature.

### 3.2. Adsorption of Zinc

To uptake zinc from single and complex solutions, Ong et al. [42] and Xu et al. [37] used thermally treated rice husk. Meretin et al. [43,44] first activated thermally treated rice husk with acetic acid, while in contrast, Akhtar et al. [38] heated rice husk after modification with chemicals. El-Shafey [45] treated rice husk with H_2_SO_4_ at 175–180 °C. Treatment with H_2_SO_4_ and CH_3_COOH appears to be effective to produce sorbents for zinc removal. RH_H2SO4wet_, RH_H2SO4dry_, and RHC_AA_ samples have the strongest affinity to zinc adsorption (Table S5). It was determined [43,45] that the pH of the initial solution decreased slightly after Zn^2+^ uptake. The release of hydrogen ions into the solution indicates the ion exchange mechanism of sorption. 

New sorbents were also active in complex solutions and in column adsorption studies at 25–50 °C (Table S6) [39,41]. Further growth of the temperature to 80 °C led to an insignificant decrease in this index [41] because the Zn (II) adsorption process is exothermic in nature [38,44]. 

In general, zinc adsorption onto rice-husk-based sorbents, depending on their production method, fits well with both Langmuir and Freundlich models and follows pseudo-first- and pseudo-second-order kinetic models and an intra-particle diffusion mechanism [37,38,39,41,42,45]. 

### 3.3. Adsorption of Copper

Bozęcka et al. [46] showed that even milled and sieved rice husk has a high enough level of sorption activity with respect to Cu^2+^ (removal percentage of 66.7% and maximum adsorption capacity of 41.1 mg·g^−1^). Alkali-treated ERH, RH_a_, and RH_NaOH_ samples have more than 10 mg·g^−1^ of maximum adsorption capacity values in the range of pH 5.5–6.5 (Table S7). The copper desorption percentage, unlike the cobalt desorption percentage, reached 88.9% (Tables S7 and S13). 

RHB, MB, EDTA-RHB, RH1, and RH_NaOH_ removed Cu (II) ions well from multi-metal systems (Table S3). All of them, except MB and EDTA-RHB, had the highest removal capability with respect to Cu^2+^ in comparison to other metal ions (Pb^2+^, Cd^2+^) coexisting in complex solutions (Tables S2–S4) [36,40]. 

Cu^2+^ adsorption onto RH_ms_, ERH, RHBC, and RH_wNcT_ was described by Langmuir isotherm models [37,38,46,47], and that onto RH_a_ fit the Freundlich equation [39]. The process fit both models in the case of MB [36]. The pseudo-first-order kinetic model and intra-particle diffusion mechanism described Cu^2+^ adsorption onto RH_wNcT_ [38], while the pseudo-first- and pseudo-second-order kinetic models and the Elovich model described this process onto MB [36]. The Cu^2+^ adsorption process was exothermic, stable, and spontaneous (ΔH° = −25 kJ·mol^−1^, ΔS° = −68 J·mol^−1^·K^−1^, and ΔG° = −3.1 kJ·mol^−1^) [38].

### 3.4. Adsorption of Chromium

Krishnani et al. [39], Khalil et al. [48], Fan et al. [49], and Pourfadakari et al. [50], studying Cr (III) and Cr (VI) sorption, emphasized the importance of pH values. The Cr (VI) sorption percentage decreased when the pH value increased above 5.2 in [48], 2 in [49], and 3 in [50] (Table S9). The efficiency of low pH values in the case of Cr (VI) sorption is explained by an enhancement of the sorbent surface positive charge as a result of protonation of carboxyl and hydroxyl groups [48,49]. This contributes to increased sorption of negative Cr (VI) through electrostatic attraction [49]. Moreover, the reduction process of hexavalent to trivalent chromium accompanied by Cr (III) adsorption onto the adsorbent surface takes place in acidic medium [39]. Cr (VI) adsorption is increased with an increase in the contact time of adsorbent-solutions. It reached a maximum in 90 min [49], 100 min [50], 120 min [48], and 96 h [39] after it became saturated. 

As the initial Cr (VI) concentration increased, the uptake of chromium increased due to an enhanced driving force [48]. The same trend was observed in [49] and the opposite trend in [50]. The increase in Cr (VI) removal from the aqueous medium with growth of the adsorbent dosage in the range of 0.2–1.5 g·dm^−3^ was fixed in [50], but it was noted in [48] that the value of this parameter passed through the maximum at 0.6 g·dm^−3^. 

The presence of selected anions affected Cr (VI) sorption onto RHP_450_ in the following sequence: SO_4_^2−^ > PO_4_^3−^ > NO_3_^−^ [48]. However, the affinity of RHC_a_-CO_2_ for chromium (III) was found to be the highest compared to Fe, Mn, Zn, Ni, and Pb [41] (Table S14). Due to this, Lattuada et al. [41] explained the insignificant impact of temperature (25, 50, and 80 °C) on the adsorption capacity of RH_Un_ and RHC_a_-CO_2_, although Fan et al. [49] observed an increase in the parameter under consideration with an increasing temperature from 25 °C to 45 °C (Table S9). The latter is understandable, since this process is endothermic [49,50]. 

According to data from [35,41,48,49,50], the chromium adsorption processes by rice-husk-based sorbents were favorable and fit the Langmuir isotherm model for Cr (VI) in the case of single solutions and the Freundlich isotherm model for Cr (III) in the case of mono- and multi-metal solutions (Tables S9 and S14). This was described by the pseudo-first-order kinetic model in [50] and the pseudo-second-order kinetic model in [48,49]. 

### 3.5. Adsorption of Nickel and Manganese

RH_a_, RH_Un_, and RHC_a_-CO_2_ sorbents (Table 1) were used to study Ni^2+^ and Mn^2+^ sorption in mono-metal solutions in batch and column experiments and in multi-metal solutions in column experiments (Tables S10 and S14), in comparison to other PTEs, such as Pb, Cr, Zn, Cd, Cu, Hg, Co, and Fe. The growth of Ni^2+^ and Mn^2+^ ions’ removal as well as other metals was observed when pH increased from 2 to 6 [39]. The maximum uptake of metals was at pH 6 due to the decline in competition between proton and metal ions. A further increase in pH values could provide metals’ precipitation (for example, in the case of nickel, it occurred at pH 10.8). In the column experiments, 100% of metal uptake was reached at low elution rates of 0.2–1 cm^−3^·min^−1^ [39]. 

These ion adsorption processes were described by the Langmuir isotherm model (Table S10), although Lattuada et al. [41] determined that while Ni^2+^ adsorption onto unmodified rice husk fit the Langmuir model, that onto rice husk carbonized and activated by KOH and then CO_2_ fit the Freundlich isotherm model (Table S14). Mn^2+^ ions’ adsorption onto both sorbents was described by the Freundlich isotherm model (Table S14). 

The maximum adsorption capacity values of nickel onto RH_a_ and RH_Un_ were the lowest among other PTEs (Tables S1, S5–S10, S13, and S14), and in contrast, manganese had the highest affinity to RH_Un_ compared with the other PTEs examined (Tables S6 and S14). The Ni^2+^ and Mn^2+^ adsorption Gibbs-free energy values were negative (Table S14), showing that the sorption processes were spontaneous, except for Mn^2+^ adsorption onto RH_Un_. In the latter case, the ΔG value was equal to 0 (Table S14), indicating that there is an equilibrium state of the system. 

## 4. Adsorption of Ferrous Metals

### Adsorption of Iron

Fe (II) and Fe (III) adsorption onto different kinds of rise-husk-based sorbents was studied by Sheveleva et al. [40], Lattuada et al. [41], and Maliki et al. [51]. According to the results of [40], the RH_s_ sample, representing silica, is the most active in the process under consideration (Table S11). Maliki et al. [51] showed that the porous system forming in the rice husk carbonization process, followed by the physical activation process, plays the main role in iron ions’ adsorption. Therefore, RHC-400-A650 was more active with respect to iron ions than RHC-400. 

RH_Un_ and RHC_a_-CO_2_ sorbents showed a high ability to uptake Fe (II) from acidic complex solutions in continuous tests. Fe (II) adsorption fits the Freundlich isotherm model. The negative calculated values of ΔG indicate that the Fe (II) adsorption process is spontaneous (Table S14) [41].

## 5. Adsorption of Minor Metals

### 5.1. Adsorption of Mercury

Rocha et al. [52,53,54] studied a rice husk activity to remove mercury from synthetic and natural water (Vouga River, Portugal) during a series of consistent experiments (Table S12). Equilibrium was achieved for a long period of time (168 h). The maximum value of mercury removal changed depending on the initial concentration of metal and was 81% and 93% in the cases of 0.05 and 0.5 mg·dm^−3^ of Hg (II) solutions, respectively. Similar Hg removal percentages were obtained in spiked ultra-pure water under identical test conditions: 84% and 92% in the remediation processes of ultra-pure water spiked with Hg (II) in quantities of 0.05 and 0.5 mg·dm^−3^, respectively [52].

Despite that the difference was not relevant, different mechanisms of Hg removal from solutions were declaimed because the water-dissolved organic materials greatly contribute to the mercury removal process as well. This thesis needs to be clarified. It is not understandable why losses (almost 50%) of Hg in river water with low levels of this pollutant [52] were the same as in the control experiments (for solutions with both Hg (II) concentrations, but without rice husk) [54] and decreased sharply (to 10%) in river water containing high concentrations of Hg [52]. Nevertheless, the undoubted advantage of natural rice husk is the possibility of reusing it with high efficiency [53] and elimination of the toxicity to the different kinds of organisms [52].

High values of the maximum sorption capacity of Hg (II) (up to 385 mg·g^−1^; Table S12) were reached in the case of using sorbents chemically prepared by H_2_SO_4_ treatment of rice husk [45] as a result of the redox process between the sorbent and Hg (II). This method was found useful to remove mercury from chloride media because it formed insoluble Hg_2_Cl_2_, unlike HgCl_2_. A decrease in Hg^2+^ removal at pH higher than 7 was observed because of mercuric hydroxide formation [39]. According to the Langmuir model, the maximum adsorption capacity of alkali-treated rice husk (RH_a_) was also high (36.1 mg·g^−1^; Table S12), second only to the same indicator for lead (58.1 mg·g^−1^; Table S1) [39]. Good results were also obtained in column experiments with single solutions at flow rates of 0.2–1.0 cm^3^·min^−1^, as well as for other metals.

In general, the Hg (II) adsorption process onto rice-husk-based adsorbents was described by the Langmuir isotherm equation and the pseudo-second-order kinetic model, except for unmodified RH_in500_ (Table S12). Most likely, this is explained neither by the method of the sorbent production nor by the low level of mercury concentration used in [52,53,54], unlike in [39,45].

### 5.2. Adsorption of Cadmium 

Kumar et al. [55], Akhtar et al. [38], and Sheveleva et al. [40] tested rice husk that was crushed and sieved or unground, washed with water, and dried under different conditions as a sorbent in mono-metal solutions, and Roha et al. [36] examined it in multi-metal solutions. RH_i_ (Table S8) exhibits the highest value of maximum adsorption among all unmodified samples in batch experiments. It has a typical composition involving cellulose (31.12%), hemicellulose (22.48%), lignin (22.34%), mineral ash (13.87%), water (7.86%), and extractives (2.33%) [55], but the physical and chemical characteristics look amazing. For example, the ash content (48.81%) and surface area (320.9 m^2^·g^−1^) values seem to be too high. 

The Cd^2+^ adsorption capacity of the examined sorbents was increased from pH 1–2 to pH 6 in [36,38], from pH 4 to pH 6 in [55], and then maximized at 6. Adsorption equilibrium was achieved in 20 min [38] and 60 min [36,55]. The percentage of cadmium removal decreased when the Cd^2+^ initial concentration increased in the ranges of 10 to 100 mg·dm^−3^ [38], 0 to 20 mg·dm^−3^ [36], and 20 to 100 mg·dm^−3^ [55]. 

Liu et al. [56], Xu et al. [37], and Akhtar [38] used rice husk after heat treatment. The longer this treatment was, the lower the maximum adsorption capacity values of RHC (18.69 mg·g^−1^), RHBC (7.8 mg·g^−1^), and RH_wNcT_ (4.95 mg·g^−1^) sorbents were in mono-metal systems (Table S8). Obviously, this can be explained by the large losses of functional groups during longer heating of rice husk. RH_a_ and RH_NaOH_ samples, obtained by alkaline treatment, exhibited similar affinity to Cd(II) in mono-component solutions, as well. Their maximum adsorption capacity values were 16.7 and 14.5, respectively [39,40]. Optimal pH was found to be 5.5–6 [39], as in the case of using unmodified rice husk. The best result was achieved using xanthate-modified rice husk (RH-X) in a single system (Table S8) [57]. RH-X provided 138.85 mg·g^−1^ of Cd (II) uptake.

No RHBC or RH_NaOH_ worked with cadmium ions in multi-metal solutions (Table S4). Unmodified RH1, carbonized RHB, chemically modified EDTA-RHB, and their mixture MB had low maximum adsorption capacity values (0.26–0.48 mg·g^−1^), although they provided 50–100% of Cd (II) removal from solutions containing Pb (II), Cu (II), and Zn (II) (Table S4). 

Krishnani et al. [39] also investigated column adsorption using RH_a_ and 12.5 mg·dm^−3^ of Cd (II) solution. RH_a_ did not provide leakage of cadmium ions up to 33 bed volumes. In general, the column capacity of RH_a_ was higher than 1 in batch experiments, and it increased when the flow rate decreased, achieving maximum removal at 0.2 cm^3^·min^−1^. It was shown [39,55] that regeneration of unmodified rice-husk-based sorbents is technically feasible but not necessary, and 97% of sorbed metals were eluted with 20 cm^3^ of 0.1 M HCl [38].

The experimental data of Cd(II) adsorption onto RH_i_, RH_a_, and SCActA fit the Freundlich isotherm model [35,39,55], but Cd^2+^ sorption onto RHC, RHBC, and RH_wNcT_ was described by the Langmuir equation [37,38,56]. The thermodynamic parameters showed that the studied process was feasible, spontaneous, and exothermic in nature [38,55]. Kinetics of cadmium sorption were described by the pseudo-second-order equation in the case of RH_i_ and RHC [55,56] and the pseudo-first-order and intra-particle diffusion equations in the case of RH_wNcT_ [38]. This explains ion exchange and complexation as mechanisms of Cd (II) sorption [37,40,57]. The possibility of Cd (II) with phenolic OH-group complexation on RHBC was confirmed by FTIR results [37].

### 5.3. Adsorption of Cobalt 

Milled rice husk (0.5 mm) was examined in the Co^2+^ adsorption process from aqueous solutions with a large range of Co^2+^ concentrations (from 10 to 10,000 mg·dm^−3^) at pH 4.0, in comparison to the synthetic ion exchanger and activated carbon [46,73]. The tested sorbent was more effective (sorption degree was >70%) in diluted (10 mg·dm^−3^) solutions, although it worked in solutions with high concentrations of cobalt (up to 10,000 mg·dm^−3^) as well. In general, the new biosorbent showed a low Co^2+^ desorption degree (28.1%; Table S13), although its activity was almost equal to the effectiveness of Norit SX2-activated carbon. 

A low percentage of Co^2+^ removal was achieved onto alkali-treated rice husk in column experiments using mono- and multi-metal solutions as well. In the case of multiple solutions, the sorption of Co^2+^ ions was affected by Pb^2+^ and Cu^2+^ ions [39]. According to the Langmuir model, the milled rice husk’s maximum sorption capacity to remove Co^2+^ ions was high enough (494.2 mg·g^−1^), unlike that for alkali-treated rice husk (9.57 mg·g^−1^) (Table S13) [39,46]. 

### 5.4. Adsorption of Arsenic

A hydrogel biochar composite (HBC-RHs; Table 1) was created to remove arsenic from wastewater [58]. The metal uptake degree was dependent on the adsorbent dosage, the initial concentration of arsenic solution, and the contact time. The optimal value of pH was 6, and the highest arsenic uptake degree, achieved for the first 6 h, was 95%. It increased to almost 100% in 24 h, although it took about 48 h to reach an equilibrium state. The arsenic adsorption process was described by the Langmuir isotherm (R^2^ = 0.999) and the pseudo-second-order kinetic models. The maximum sorption capacity calculated using the Langmuir model was 28.32 mg·g^−1^ (Table S15).

A hybrid adsorbent (RHIOB; Table 1) involving two active phases (biochar and iron oxide) provided a degree of high arsenic removal (90%; Table S15) from solutions with low (0.05–0.2 mg·dm^−3^) concentrations of this pollutant in 24 h [59]. The main adsorbent phase of As (III) was iron oxide (Fe_3_O_4_ and Fe_2_O_3_) due to the chemisorption that occurred with the formation of Fe-O-As(III) groups. The 6.5–7.5 pH range was preferable for arsenic removal by the tested sorbent, although this process was run in a wide (2.0–10) pH range. The Langmuir maximum capacity of the sorbent was 0.096 mg·g^−1^ (Table S15). 

A hybrid iron oxide–biochar composite well-adsorbed As (III) from single systems including individual chloride, nitrate, sodium, potassium, and calcium, with concentrations of 100–200 mg·dm^−3^. The presence of sulfate, bicarbonate, and especially phosphate in the same ranges of concentrations affected arsenic adsorption from mono-metal systems. The mixture of the above-mentioned impurity ions, except phosphate, in multi-metal solutions did not significantly decrease As (III) adsorption. The advantage of this sorbent was that its regeneration and reusability were at a minimum during four cycles, without a sharp drop (from 90% to 86%) of adsorbent efficiency (Table S15). 

The process of As (III) adsorption onto RHIOB was best described by the Redlich–Peterson equation at 45 °C. As the temperature rose, the value of the maximum adsorption capacity of arsenic increased, indicating that As (III) adsorption was an endothermic process. It fit the pseudo-second-order kinetic model. Particle and film diffusion were the rate-limited stages at low (0.05 mg·dm^−3^) and high (0.1–0.2 mg·dm^−3^) concentrations of arsenic, respectively.

## 6. Adsorption of Radionuclides

### 6.1. Adsorption of Cesium and Strontium

Powder-TiSi and carbon-TiSi fractions (Table 1) were used to remove radionuclide ions [60]. Cs^+^ and Sr^2+^ ions’ adsorption processes from a 0.01 M NaCl water solution by these biochars, with titanium silicates precipitated on their surfaces, were carried out in batch experiments at a solid (g) to liquid (cm^−3^) ratio of 1:50 at room temperature for 48 h, with stirring. Autoclave powder-TiSi was more active in the cesium adsorption process (Cs^+^ ions’ distribution coefficients K_d_ = 27,000) than powder-TiSi obtained by water vapor blowing at different temperatures (K_d_ = 600 ÷ 1200). However, it was worse in comparison to known adsorbents for this isotope. The powder-TiSi obtained by blowing water vapor at 400 °C was the best adsorbent for the stable strontium isotope (K_d_ = 2,095,000). Unfortunately, this phenomenon was not explained, nor was the behavior of the carbon-TiSi fraction. These questions need to be clarified. 

### 6.2. Adsorption of Uranyl Ion

Expanded rice husk powder was used to uptake uranyl ions from aqueous medium by Zhang et al. [61]. Tests were carried out under static conditions with different parameters of the process, such as the pH, adsorbent–solution contact time, initial concentrations, and adsorbent dosage. The maximum uranium adsorption capacity was found to be 5.7 mg·g^−1^ from 80 mg·dm^−3^ at pH 3. As the initial concentration decreased, so did the uranium adsorption capacity. Depending on the adsorbent dosage, it was possible to reach 90% of the removal efficiency. The adsorption process is described well by the Langmuir model. As for an adsorption mechanism, the chemisorption, ion exchange, and physical sorption processes were demonstrated to take place. According to the obtained data, uranium adsorption on expanded rice husk fits the pseudo-second-order kinetic model.

## 7. Adsorption of Precious Metals 

### 7.1. Adsorption of Silver 

Luo et al. [62] and Liang et al. [63] investigated Ag^+^ ions’ adsorption using rice husk and expansion-treated rice husk powder produced by Haitian High-Tech Material Co., Ltd., without added purification, respectively. In [62], the effects of the pH (1–6), adsorbent mass (0.1–1.3g), initial silver concentration (50–2000 mg dm^−3^), and contact time (1–135 min) have been studied during batch experiments (volume Ag^+^ solution—100 cm^−3^, stirring speed—180 rpm, and temperature—25 °C). The greatest Ag^+^ removal percentage (96.13%) by 0.5 g of rice husk from a 10 mg·dm^−3^ silver solution was at pH 1, and its lowest value (51.92%) was at pH 4. The pH values after the adsorption increased as a result of competitive adsorption of Ag^+^ and H^+^. The lower the Ag^+^ removal efficiency, the more the pH increases. 

The effect of the adsorbent mass used to remove silver ions from solution was the same for the same kind of process. The Ag^+^ removal percentage rose sharply from 63.13% to 90.81% when the adsorbent mass increased from 0.1 g to 0.3 g at pH 2 and a silver concentration of 10 mg·dm^−3^. However, when the quantity of rice husk powder was more than 0.3 g, the silver removal efficiency grew slowly, reaching a maximum of 98.8% at 1.3 g of adsorbent, because the equilibrium between the concentrations of silver ions in the solution and on the surface of the sorbent was reached. This equilibrium was reached very quickly (in 20 min). 

According to the calculated kinetic data, the process was described by the pseudo-second-order kinetic model, i.e., the rate-limiting step was chemical adsorption. The studied adsorption process is described well by the Langmuir model [62]. The same result was found in [63]. The maximum adsorption capacity was 42.43 mg·g^−1^ in [62] and 18.6 mg·g^−1^ in [63]. Therefore, both natural and expansion-treated rice husks are good materials to sorb silver ions.

### 7.2. Adsorption of Gold

Aktas and Morcali [64] and Morcali et al. [65] showed that activated rise husk (ARH; Table 1) was an active adsorbent, similar to the chelating resin Lewatit TP-214, with respect to gold ions. The quantity of gold adsorbed in batch experiments increased with the increasing temperature and contact time. Both sorbents demonstrated a maximum ability to sorb gold under acidic media (pH = 2). The percentage of gold removal dropped when the solution pH increased. Depending on the process conditions, the gold uptake by both sorbents was 100%. 

The calculated value of activation energy for gold removal by ARH (28.44 kJ mol^−1^) was a slightly greater in comparison to that of Lewatit TP-214 (26.41 kJ mol^−1^) [64]. These values of activation energies indicate that the studied processes are controlled by mixed mechanisms, i.e., they are intermediate-controlled processes [64]. The gold adsorption processes onto the tested sorbents were described by Langmuir equilibrium [65]. The maximum adsorption capacity of gold ions at 25 °C onto ARH was 93.46 mg·g^−1^, and that onto Lewatit TP-214 was 108.70 mg·g^−1^. According to the thermodynamic constants calculated, the adsorption processes were found to be endothermic and spontaneous. 

Carbonized and activated rice husk was applied by Mansurov et al. in the electrochemical sorption/desorption Au^3+^ ions’ processes [66,74]. The carbon-nanostructured adsorbent showed the highest gold removal at pH 2. Moreover, it demonstrated a high selectivity to gold ions in the combined presence of copper, nickel, and silver. The optimal flow rate of solutions during the electrochemical reduction sorption of gold was set up as 10 cm^3^·min^−1^. It was established that the desorption process was running effectively (96% recovery) in an acetone/water/NaOH mixture. 

### 7.3. Adsorption of Palladium

RH@MCM-41@ARS (Table 1) was used as a sorbent for separation and preconcentration of Pd^2+^ from different samples [68]. The optimum parameters of the Pd^2+^ ions’ removal process from aqueous solution with a Pd^2+^ concentration of 10 mg·dm^−3^ were determined as follows: pH 4, 20 min of stirring time, and 50 mg of adsorbent mass per 1000 cm^3^ of solution volume. Palladium ions’ removal was >95% under these conditions and the preconcentration factor was almost 200 when 5 cm^3^ of 0.5 mol·dm^−3^ thiourea solution was used as an eluent (98.6% recovery). According to the Langmuir model (the correlation coefficient for the adsorption, R^2^, was 0.998), the adsorption process was favorable, and the maximum adsorption capacity of Pd^2+^ onto RH@MCM-41@ARS was 198.2 mg·g^−1^, i.e., it was much higher than that onto RH@MCM-41 (16.1 mg·g^−1^) and RH (13.3 mg·g^−1^). 

RH@MCM-41@ARS showed selective Pd^2+^ adsorption in the presence of different kinds of ions. Tolerance limits of interfering ions in the determination of 0.1 mg·dm^−3^ Pd^2+^ were found for the following cations and anions (mg·dm^−3^): Na^+^, Mg^2+^, Ni^2+^, K^+^, and SO_4_^2−^—4000; Co^2+^, Cr^3+^, Br^−^, SCN^-^, HCO_3_^−^, and Cd^2+^—1000; Ba^2+^ and Pb^2+^—500; Cu^2+^ and Fe^3+^—250; Rh^3+^—50; Pt^4+^—10. The efficiency of RH@MCM-41@ARS use was kept constant after 10 adsorption/desorption cycles. The precision of Pd^2+^ determination in spiked samples of seawater, Nile water, wastewater, and clay after separation and preconcentration using RH@MCM-41@ARS was 98.0–99.4%.

## 8. Adsorption of Rare and Rare-Earth Metals

### 8.1. Adsorption of Lanthanum and Erbium

For lanthanum and erbium removal from aqueous media in water purification, or for the treatment of industrial wastewater, Awwad et al. [69] tested rice husk activated by H_3_PO_4_ and then carbonized at 700 °C for 2.5 h. The sorbent had the following surface properties: a specific surface area of 451.82 m^2^·g^−1^, a total pore volume of 0.3437 cm^3^·g^−1^, and an average pore radius of 1.52 nm. Batch experiments were carried out using 0.03 g of the adsorbent with 10 cm^3^ of solution containing 50–300 mg·dm^−3^ of La (III) or Er (III). Maximum recovery of these metals was achieved at pH 4. 

The modified rice-husk-based adsorbent had a high adsorption capacity, such as 175.4 mg·g^−1^ for La (III) and 250 mg·g^−1^ for Er (III). Both the Langmuir and Freundlich isotherm models well described lanthanum (R^2^ = 0.981 and 0.987, respectively) and erbium (R^2^ = 0.981 and 0.994, respectively) sorption processes on the rise-husk-based adsorbent. Obviously, external and intra-particle diffusion limited the adsorption rate in both cases. The kinetics of sorption were described by the pseudo-second-order model.

### 8.2. Adsorption of Cerium

Rice husk washed with water and dried without other treatment was examined for removal of Ce (IV) from aqueous solution [70]. Ce (IV) adsorption onto rice husk was confirmed by Fourier transform infrared spectroscopy (FTIR) and energy-dispersive X-ray (EDX) analysis. Optimal conditions of adsorption were determined as follows: 25 min of contact time and 0.25 g of sorbent per 4 cm^3^ (62.5 g·dm^−3^) of cerium ions solution. The percentage of Ce (IV) removal decreased from about 80% to almost 35% when the initial metal concentration increased from 5 to 60 mg·dm^−3^. The opposite trend was observed with the increasing adsorption temperature. When it increased in the range of 5–60 °C, the percentage of sorption of Ce (IV) increased from ~69% to ~79%. The maximum adsorption capacity was 0.55 mg·dm^−3^. 

This begs the question of why the authors concluded that the adsorption data fit the Langmuir isotherm model, even though the linear and non-linear forms of the Freundlich and Dubinin–Radushkevich models also described the investigated process well enough. Moreover, the regression coefficient (R^2^) of the Freundlich isotherm linear form showed the highest (0.993) value compared to the R^2^ values of the Langmuir (0.9872) and Dubinin–Radushkevich (0.980) models. 

As for the adsorption kinetics, it fit the pseudo-second-order model. Ce (IV) sorption onto rice husk was characterized as an endothermic and spontaneous process according to the thermodynamic results, such as: ΔH = 8.65 kJ·mol^−1^, ΔS = 38.2 J·mol^−1^·K^−1^, and ΔG_280 K_ = −10.7 kJ·mol^−1^ (ΔG_332 K_ = −12.7 kJ·mol^−1^).

### 8.3. Adsorption of Rhenium

Activated rice-husk-based biochars were tested to remove Re (VII) ions from single solutions at pH~6 in batch experiments for different periods of time [71] (Table S16). The adsorption equilibrium was reached fast enough (in 30 min) and the percentage of Re (VII) extraction was high (~90%). The initial concentration of the metal solution increased (20–100 mg·dm^−3^), and the maximum adsorption capacity value of Re (VII), depending on the adsorbent–adsorptive contact time (30–60 min), trebled, reaching at least 8.8 mg·g^−1^ [71]. The percentage of Re (VII) removal was higher for 60 min (96%) compared with 30 min (84%), and it dropped more significantly (from 84% to 32%) in the 30 min process when the initial concentration of Re (VII) increased [71]. Based on the Giles isotherm classification [75], Kablanbekov et al. [71] classified the isotherm of Re (VII) removal for 60 min as the C curve considering that the number of sites on the KHC4-600VA surface for Re (VII) adsorption remained constant at all concentrations up to saturation. The Re (VII) adsorption process onto KHC4-600VA was described the best by the Freundlich equation, while in the case of SCActA, it fit well with the Langmuir isotherm model (Table S16). Adsorption of Re (VII) ions took place favorably onto both sorbents, which had a strong affinity to the sorbed ions [35,71].

To study this process, Zharmenov et al. [72] also used a solution produced in the hydrometallurgical shop of the JSC PS “Yuzhpolymetall” containing 35 mg·dm^−3^ of Re (VII). Batch and column experiments were carried out. The Re (VII) uptake from an industrial multi-metal solution by RH_NaOH-S-gr_ was 75%. Re (VII) ions were absent in 70 bed volumes. The column effluent concentration attained an initial concentration after 244 bed volumes. Using an 8% NH_4_OH solution as the eluting agent, it was possible to concentrate this rare metal 18 times [72].

## 9. Assessment of the Effectiveness of Rice-Husk-Based Adsorbents Depending on Production Methods

**Figure 1:**
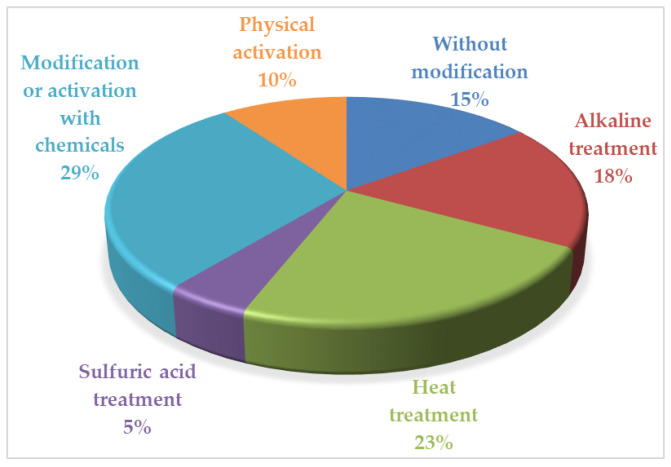
Share of the use of various methods for the RH-based adsorbents’ production.

The adsorption activity of rice-husk-based adsorbents depends on many factors. First is the preparation method because it provides their properties and behavior in adsorbtive media. Since rice husk contains of a large quantity of silicon dioxide (~14%), increasing after its heat treatment (the most common stage in the RH-based sorbents’ production; Figure 1), alkaline treatment is a very popular method as well. 

Based on the results of the analysis of rice husk and its derivatives by infrared spectroscopy, scanning and transmission electron microscopy, and temperature-programmed desorption mass spectrometry [18,71,76], it can be concluded that alkali affects raw and heat-treated plant materials in different ways. Alkaline treatment of raw rice husk causes not only silicon removal but also a delignification of biomass. As a result of the cleavage of lignin, hemicellulose and cellulose bonds, and the removal of silicon, first, the specific surface area of the material increases because its swelling in aqueous solution increases. Second, defective spots are formed on the surface of the rice husk, which are potential active adsorption sites. Therefore, silicon is present in raw biomass as a hydrated form connected with biomolecules [18,76]. Consequently, after silicon removal, a negative charge accumulates on the rice husk surface, and the possibility of attracting positively charged metal ions from aqueous solution grows.

In the case of heat treatment of rice husk at temperatures above 500 °C under an O_2_-limited atmosphere or N_2_ flow, thermal destruction of biomass, forming a graphite-like structure, takes place, anhydrous silica forms, and silica−biomolecules bonds are destroyed, in general. The supply of an inert gas enhances the process of biomass thermal destruction due to the accelerated removal of forming volatile compounds from the reaction zone. Removal of anhydrous silicon dioxide under the action of alkali leads to the formation of (i) a porous system and (ii) free radicals (in places where remaining C−SiO_2_ bonds are broken) [18]. Due to this, the specific surface area and adsorption properties of heat-treated rice husk enlarge after treatment with alkali [35]. Moreover, the resulting silicate solution can be used to obtain silicate glue or high-purity fine silica. 

**Figure 2:**
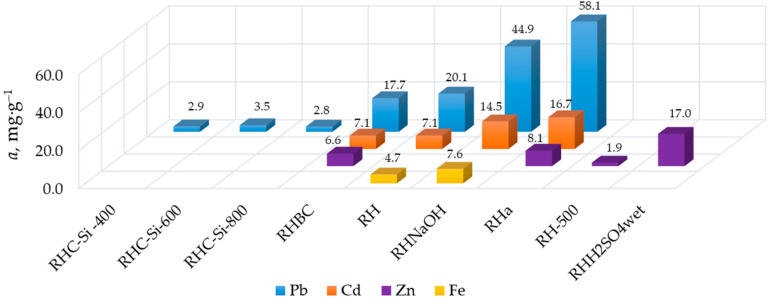
RH-based sorbents’ adsorption activity characteristics for removal of metals from mono-metal solutions in batch experiments.

Advantages and disadvantages of alkaline and heat treatments of rice husk can be concluded from the adsorption activity of sorbents, as presented in Figure 2. As can be seen in this diagram, alkali-treated rice husk (RH_NaOH_, RH_a_) has the highest values of maximum adsorption capacity with respect to various metals. Heat treatment up to 350 °C is acceptable to produce sorbents (RHBC sample), but further increases in temperature impairs their adsorption activity (RH_C-Si_-400, RH_C-Si_-600, RH_C-Si_-800, and RH_500_ samples; Figure 2). 

**Figure 3:**
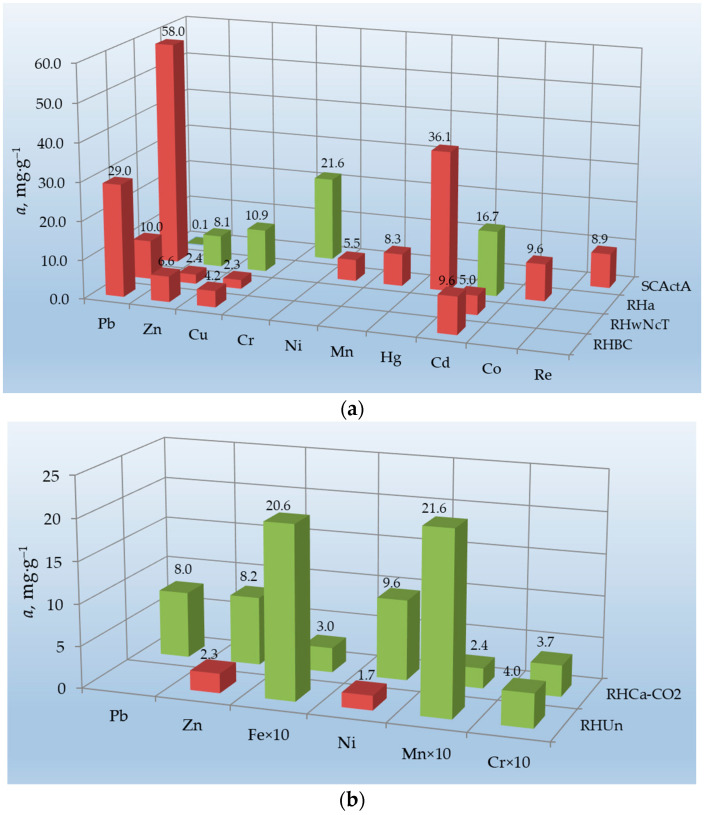
The influence of RH-based sorbents’ production methods on their ability to uptake metals from: (**a**) mono-metal solutions, batch experiments, and (**b**) multi-metal solutions, column experiments. Red bars fit the Langmuir equation and green bars fit the Freundlich equation in both diagrams.

Vapor–gas activation of rice husk carbonates treated with alkali by water vapor or carbon dioxide provides some positive results. However, it should be considered that activation with steam gases is carried out at very high temperatures (800–850 °C). Therefore, it is a very expensive process to obtain sorbents using rice husk, but no significant jump in their adsorption activity is observed. The sorbents obtained by this method (SCActA and RHC_a_-CO_2_) are inferior in their ability to sorb some metals in comparison to rice husk treated with alkali (RH_a_), carbonized rice husk (RHBC), and even unmodified rice husk (RH_Un_), as can be seen in Figure 3. 

A similar conclusion can be drawn regarding the rice husk modification with chemicals. For instance, the RH_wNcT_ sample produced using a complex multi-step process (Table 1) is not a better sorbent for Pb, Zn, Cu, and Cd removal compared to RH_a_ or RHBC (Figure 3a). At the same time, there are successful examples of sorbent production using treatment with various chemicals. Therefore, RH@MCM-41@ARS, RHC_f_-Mag-2, and RH_TA_ (Table 1) have very high values of maximum adsorption capacity: 198.2 mg·g^−1^ of Pd (II), 150.83 mg·g^−1^ of Cr (VI), and 93.45 mg·g^−1^ of Pb (II), respectively (Tables S1 and S9). 

As can be seen in Figure 1, the treatment of rice husk with sulfuric acid is an independent method for the RH-based sorbents’ production. In different studies, this process is believed to be carbonization or hydrolysis [33,45,57]. It is necessary to highlight that a product of rice husk treatment with diluted or concentrated sulfuric acid in an autoclave or concentrated sulfuric acid at room temperature is lignin. It is known that hydrolysis lignin is a good sorbent due to the presence of active oxygen containing functional groups [76]. Obviously, their quantity increases after alkaline treatment. The H_2_SO_4_-treated RH-based sorbents are the best for removal of Pb (RH_C-OX_; Table S1), Cd (RH-X; Table S8), Hg (RH_H2SO4 wet_; Table S12), and Zn (RH_H2SO4 wet_; Figure 2, Table S5). The problem is the very aggressive conditions for obtaining them.

**Figure 4:**
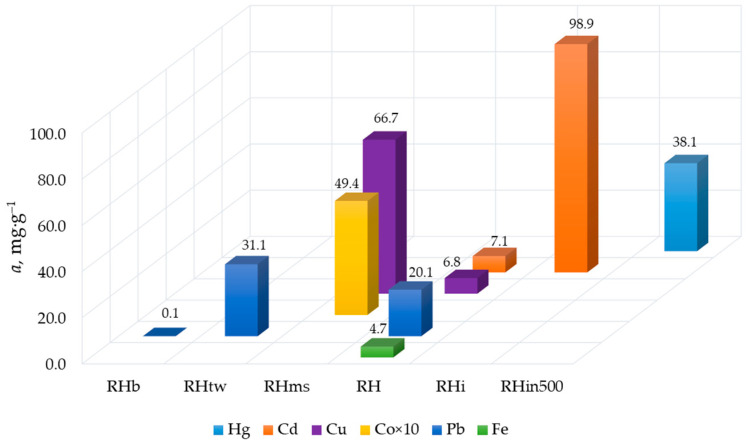
Adsorption activity of unmodified rice husk to remove various metals from mono-metal solutions in batch experiments.

As for unmodified rice husk, as mentioned above, it removes many kinds of metals well from aqueous solutions. Milling obviously plays the main role in preparing raw rice husk for use in sorption processes. The smaller the grain size, the higher the sorption capacity of the rice husk. For example, RH (usual size of rice husk is 5 mm and larger) was used without grinding (Table 1). Its adsorption capacity is significantly lower than RH_tw_ (100–200 μm) toward Pb, RH_ms_ (0.5 mm) toward Cu, and RH_i_ (crushed) toward Cd (Figure 4). Prolonged boiling in water (RH_b_ sample; Table 1) negatively affects the sorption properties of rice husk (Figure 4). It may not be entirely correct to consider rice husk milled and boiled in water as unmodified. It is widely known that mechanical activation improves the adsorption properties of materials and boiling in water allows for removing water-soluble substances from plant materials. In the first case, new active sites are formed due to the disruption of bonds as a result of mechanical damage to plant material. In the second case, on the contrary, potential adsorption sites are removed.

For practical use, selectivity is an important property of the sorbent. The majority of RH-based sorbents discussed in this paper were not characterized as selective sorbents, or this parameter was not studied for them at all. In the case of mono-metal solutions, the affinity of the same sorbent for metal ions in batch experiments increases mainly as their ionic radii increase (nm; Figure 2 and Figure 3a): Pb^2+^(0.113) > Hg^2+^ (0.103) > Cd^2+^ (0.090; 0.102 in solution) > Mn^2+^ (0.083; 0.080 in solution) > Fe^2+^ (0.076) *≥* Zn^2+^ (0.076; 0.070 in solution) > Cu^2+^ (0.075; 0.072 in solution) *≥* Co^2+^ (0.074; 0.072 in solution) > Ni^2+^ (0.069; 0.067 in solution) > Fe^3+^ (0.062; 0.064 in solution) > Cr^3+^ (0.061; 0.058 in solution) > Re^7+^ (0.053) [77]. 

The rule is violated in multi-metal solutions, especially in column experiments (Figure 3b). In complex-component solutions, competitive sorption occurs. Its result is influenced by many factors, in addition to the ionic radius (or ionic potential in general). As can be seen in Figure 3b, the sorption of metals with unfilled 3d orbitals (Mn, Fe, and Cr) is the highest. It is likely that these metals are capable of forming more stable complexes with functional groups of sorbents (firstly carboxylic), in particular rice husk (RH_Un_). The adsorption values of these metals onto RHC_a_-CO_2_ decrease, but the general trend of changing them is largely consistent with that of RH_Un_. This can be explained by the decrease in the number of functional groups capable of complexation after heat treatment of rice husk at 300 °C, followed by alkaline treatment and activation with carbon dioxide at 780 °C (RHC_a_-CO_2_; Table 1). This is in good agreement with the heterogeneous nature of sorption described by the Freundlich equation that takes place in complex solutions (green bars in the diagram in Figure 3b), in contrast to single solutions, in which homogeneous adsorption described by the Langmuir equation takes place (red bars in the diagram in Figure 3a).

## 10. Prerequisites, Current Status, and Tasks for the Future

Research on the processing of rice husk as raw materials to produce adsorbents for various purposes has a wide geography. Scientists from scientific organizations and universities in China, India, the USA, Canada, Pakistan, Iran, Malaysia, Turkey, Russia, Poland, Oman, Portugal, Nigeria, Saudi Arabia, Egypt, Kazakhstan, Brazil, Indonesia, Taiwan, Italy, and other countries are mainly working in this area. The great interest in this issue demonstrates its global nature and relevance. The prerequisites for the work under consideration were: (i) the need to solve the environmental problem of recycling multi-tonnage waste from rice production; (ii) ensuring high quality of water resources using adsorption processes that are less costly, easy to perform, and effective; (iii) creating affordable adsorbents as an alternative to expensive commercial sorbents for cleaning water bodies from various pollutants; (iv) development of competitive methods for obtaining effective adsorbents for the extraction of metals from the aqueous environment. Various methods of making sorbents from rice husk can be categorized into the following main approaches:

- The use of raw rice husk: unmodified, washed with water, and dried—crushed or non-crushed [28,29,36,38,40,41,46,52,53,54,55,70].
- Modification and activation of rice husk by the action of organic and/or inorganic compounds [30,31,32,33,34,36,38,39,40,43,44,47,49,50,57,58,59,61,62,63,68,69,71].
- Alkaline treatment of rice husk, its derivatives, and carbonates [30,31,35,39,40,41,47,49,50,60,66,67,71,72].
- Treatment of rice husk by sulfuric acid [33,45,57].
- Heat treatment of raw or modified rice husk [34,36,37,40,42,43,44,48,49,51,56,59,60,64,65,66,67,69].
- Products of rice husk processing physical activation [38,41,64,65,66,67,71,72].

Sorbents based on rice husk have been tested in the adsorption processes of non-ferrous [28,29,30,31,32,33,34,35,36,37,38,39,40,41,42,43,44,45,46,47,48,49,50], ferrous [40,41,51], minor [35,36,37,38,39,40,45,46,52,53,54,55,56,57,58,59], radioactive [60,61], precious [62,63,64,65,66,67,68,74], rare, and rare-earth metals [35,69,70,71,72]. The main task solved during the research was the purification of wastewater and natural objects from toxic metals. Preconcentration and concentration of metals in hydrometallurgical processes is another important task, albeit less common. The vast majority of studies were carried out using artificially prepared solutions. Few studies have used natural water or natural water containing industrial wastewater, but both artificially polluted and original industrial wastewater have been used [38,52,54,68,69]. Even fewer used industrial solutions obtained from operating enterprises [72]. The studies performed during batch experiments with mono-metallic solutions are predominant. Column experiments are carried out quite rarely [39,41,51,72], especially in terms of studying adsorbate desorption for the purpose of concentrating [72]. There are few tests to evaluate the use of sorbents in complex metal systems [36,37,40,41,45], as well as the possibility of reuse of new sorbents [28,32,33,38,39,46,50,51,52,53,54,59,72]. There is a lack of environmental and economic studies of proposed sorbent production methods, many of which involve the use of large quantities of expensive and aggressive chemicals. The methods for processing sorbents after metals’ sorption remain undisclosed.

The research findings indicated that the adsorption efficiency of new sorbents is affected by various factors, including their preparation method, amount used, type of adsorbtive and its initial concentration, the pH and temperature of the media, and the adsorbent–solution contact time. In general, the synthesized sorbents showed a high adsorption efficiency in relation to the above-mentioned groups of metals in a wide range of adsorbtive concentrations. This confirms the promising nature of the research direction under discussion. 

The process of metals’ adsorption on the surface of different types of rice-husk-based sorbents is mainly characterized as feasible, favorable, and spontaneous. Depending on the nature of the sorbent and the adsorptive, it is exothermic or endothermic and proceeds with a decrease or, extremely rarely, with an increase [25,29,30] in the ordering of the system. The kinetics of adsorption are described by the pseudo-first- and pseudo-second-order, intra-particle diffusion, and Elovich equations. The adsorption isotherms on the same sorbent, depending on the nature of the metal, correspond to different models, dominated by Langmuir and Freundlich models. Physical adsorption, electrostatic interaction, ion exchange, and complexation are distinguished among adsorption mechanisms. Calcium and magnesium cations and carboxyl and hydroxyl groups present on the surface of sorbents are responsible for ion exchange. At a high pH (>4), as a result of deprotonation, the latter form negatively charged sites that retain metal cations due to electrostatic interaction and complexation [39].

Despite the many advantages of using rice husk as a ready-made adsorbent or lignocellulosic raw material to produce adsorbents (availability and renewability of biopolymeric raw materials, low cost, high adsorption capacity and affinity for different adsorbtives, possibility of regeneration and reuse, and lack of ecotoxicity), the existing gaps in the study of this issue become apparent. Studies are developing; however, the problems mentioned in [78] ten years ago remain. Any scientific research, whether short or long term, should be orientated toward economic output. The profitability of practical applications ultimately determines the viability of any technology or product.

In this regard, it seems appropriate to conduct further research in the area under discussion, focusing on real industrial production needs. It is necessary to strengthen column experiments, mainly on complex solutions, in order to examine the selectivity of the proposed sorbents and develop new methods for the production of selective sorbents for multi-metal systems. The study of sorption in organometallic systems is of independent interest from the point of view of hydrometallurgical processes involving extraction of metals with organic solvents, followed by sorption separation of components. Any information about using rice husk in these types of studies is absent in the literature. 

The study of adsorbate elution, development of sorbent regeneration and its reuse, and sorbent testing in large-scale trials are the components of the process of creating an effective sorbent, which require in-depth study and economic calculations. It is of scientific interest to find an explanation for the phenomenon of the deterioration of sorption properties with the development of the specific surface area and porous structure of the sorbent. A full cycle of complex research is required to make an objective conclusion on the feasibility of production and application of rice-husk-based sorbents. 

## 11. Conclusions

The performed studies have demonstrated the potential use of unmodified and processed rice husk as adsorbents for the extraction of non-ferrous, ferrous, minor, radioactive, precious, rare, and rare-earth metals from aqueous solutions. Grinding is an effective way to improve the adsorption properties of raw rice husk. Heat treatment at temperatures above 350 °C has the opposite effect, requiring additional physical or chemical activation. Rice husk modification with chemicals, as well physical and chemical activation of processed rice husk, provide an increase in the adsorption capacity of end sorbents to some metals, but are neither eco-friendly nor cheap. Due to the high silica content, alkaline treatment of raw rice husk and its carbonates is a common method of high-quality rice-husk-based adsorbents’ production.

Despite certain successes in this area of study, there are still unresolved issues. It is necessary to conduct further research aimed at creating an effective method of sorbent production based on this type of agrolignocellulose waste, with subsequent use in wastewater treatment processes from a wide range of pollutants and preconcentration or concentration of metals in industrial solutions.

## Figures and Tables

**Table 1:** Production conditions of rice-husk-based adsorbents.

Sorbent	Production Conditions	Ref.
RH_b_	RH, boiled for 6 h, washed with distilled water, dried at 105 °C, sieved to a particle size of 250–350 μm.	[28]
RH_tw_	RH, washed with tap water, then with Milli-Q water, dried at 80 °C overnight, ground, and sieved. Particles of 100–200 μm in size were used.	[29]
RH_g_	RH, washed with distilled water, and refluxed in 3, 6, 9, and 12 M NaOH solutions with the addition of ethylene glycol for 4 h at 198–200 °C. Unreacted ethylene glycol was removed by distillation. Then, 25% NH_4_NO_3_ solution was added to pH 8.0. The prepared gel was filtered and dried in air at room temperature.	[30]
RH_HP_	RH, dried, ground into 20–30 mesh, treated with 0.1 N NaOH at 23 °C for 1 h with stirring, washed with distilled water, mixed with 0.1, 0.3, 0.6, 0.9, 1.2, and 1.5 M H_2_O_2_ at a 1.0 (g):7.0 (cm^3^) ratio, then washed, and dried overnight.	[31]
RH_TA_	RH, modified with C_4_H_6_O_6_ at a 1:12.5 weight ratio for 24 h at 50 °C, heated at 180 °C for 15 min, dried under vacuum for 48 h, and mixed with poly(MMA-co-MA) at a weight ratio of 0.5, 0.75, and 1. The mixture was refluxed for 3 h using inert gas and stirring. The prepared crosslinked nanoparticles were filtered, washed with tetrahydrofuran, and dried at 80 °C in a vacuum oven for 12 h.	[32]
RH_C-OX_	RH, washed with distilled water, dried at 100 °C overnight, ground into 60 mesh, mixed with 70% H_2_SO_4_ at a 1.0 (g):10.0 (cm^3^) ratio for 10 min, then poured into a stainless-steel autoclave, and carbonized at 100 °C for 6 h. The residue was filtered, washed with distilled water, dried at 100 °C for 24 h, and treated with (NH_4_)_2_S_2_O_8_ in 1 M H_2_SO_4_ at a 1:10 solid-to-liquid ratio for 12 h at room temperature. The solid residue was filtered, washed with distilled water, dried at 100 °C for 12 h, and ground into a powder.	[33]
RH_C-Si_-400	RH, washed with distilled water, ground into 63 μm, soaked in 0.1 M HNO_3_ for 24 h, filtered, washed with distilled water, dried at room temperature, and carbonized in a Fixed-Bed Reactor Unit under an air-free atmosphere at 400 °C.	[34]
RH_C-Si_-600	RH, washed with distilled water, ground into 63 μm, soaked in 0.1 M HNO_3_ for 24 h, filtered, washed with distilled water, dried at room temperature, and carbonized in a Fixed-Bed Reactor Unit under an air-free atmosphere at 600 °C.	
RH_C-Si_-800	RH, washed with distilled water, ground into 63 μm, soaked in 0.1 M HNO_3_ for 24 h, filtered, washed with distilled water, dried at room temperature, and carbonized in a Fixed-Bed Reactor Unit under an air-free atmosphere at 800 °C.	
SCActA	RH, heated in a rotary furnace at 400 °C under an off-gas atmosphere for 30 min, cooled without air to room temperature, activated with water vapor at 850 °C for 30 min, and treated with 70 g·dm^−3^ of NaOH at 70–80 °C for 2 h.	[35]
RH1	RH, washed with fresh and then distilled water, air-dried for 5 days, and sieved (2 mm).	[36]
RHB	RH1, carbonized at 500 °C for 2 h under an O_2_-limited atmosphere.	
EDTA-RHB	RHB, soaked by 0.05 M EDTA (1 g:7 cm^3^), dried at 50 °C overnight, washed with 20 cm^3^ of distilled water, and dried at 50 °C for 6 h.	
MB	RH:RHB:EDTA-RHB ratio of 1:1:1.	
RHBC	RH, heated under an O_2_-limited atmosphere at 350 °C for 4 h, ground, and sieved (0.5 mm).	[37]
RH_w_	RH, crushed, sieved (100–1000 μm), washed with tap water, then washed and boiled in double-distilled DIW, and oven-dried at 110 °C for 24 h.	[38]
RH_wN_	RH_w_, soaked in 0.1 M HNO_3_ for 2 h, and oven-dried at 110 °C for 2 h.	
RH_wNc_	RH_wN_, impregnated with 1 M K_2_CO_3_, and oven-dried at 110 °C for 24 h.	
RH_wNcT_	RH_wNc_, heated at 100–200 °C at a heating rate of 10 °C·min^−1^ for 8 h under an N_2_ flow of 500 cm^3^·min^−1^.	
RH_a_	RH, subjected to 1.5% alkali treatment (300 g in 1 dm^3^), autoclaved at 121 °C for 30 min, washed with deionized water, and dried at 50 °C.	[39]
RH	RH, washed with water, and dried at 105 °C.	[40]
RH_HCl_	RH, treated with 0.1 M HCl at 90 °C for 1 h, and washed with distilled water.	
RH_s_	RH_HCl_, oxidized at 300 °C and then at 600 °C.	[40]
RH_NaOH_	RH, treated with 1 M NaOH at 90 °C for 1 h, and washed with distilled water.	
RH_Un_	RH, unmodified (information about preparation is absent).	[41]
RHC_a_-CO_2_	RH, carbonized at 300 °C for 1 h, soaked in a KOH solution at S:L ratio of 1, and activated at 780 °C for 1 h while CO_2_ was poured in for 0, 15, 30, and 60 min.	
RH-500	RH, crushed, sieved (2 mm), washed with ultra-pure water several times, oven-dried at 105 °C for 24 h, and heated at 500 °C for 2 h.	[42]
RHC_AA_	RH, washed with water, dried at 105 °C, heated at 450–500 °C for 8–10 min, cooled in distilled water (S:L = 1:5), filtered out, and treated with 2% CH_3_CHOH. Solid residue washed with double-distilled water, dried at 100–150 °C, and crushed.	[43,44]
RH_H2SO4wet_	RH, washed with distilled water, air-dried, treated with 13 M sulfuric acid at S (g):L (cm^3^) of 20:100 at 175–180 °C for 20 min with occasional stirring, cooled, washed, stored under acidic conditions (pH 1.5–2), and washed with a stream of distilled water between two sieves of 16 and 60 mesh before experiments.	[45]
RH_H2SO4dry_	RH_H2SO4wet_, dried at 120 °C.	
RH_ms_	RH, milled and sieved (0.5 mm).	[46]
ERH	RH, expansion-treated, and treated with an alkaline solution.	[47]
RHP_450_	RH, dried in the sun for 48 h, oven-dried at 65 °C for 72 h, ground in a mechanical grinder, sieved with a pulverized sieve of size < 250 µm, pyrolyzed at 450 °C for 2 h, cooled, and sieved (<250 µm).	[48]
RHC_f_	RH, powder, mixed with 2 M NaOH at S:L = 1:7, heated to 100 °C for 4 h, washed with DIW, and dried at 120 °C overnight.	[49]
RHC_f_-Mag-2	RHC_f_ (5 g), mixed with 10.0 g of FeCl_3_·6H_2_O in 50 cm^3^ of ethanol, stirred for 2 h, kept in a water bath at 50 °C to evaporate ethanol, oven-dried at 100 °C for 24 h, heated at 800 °C under N_2_ atmosphere for 2 h, ground, washed with DIW, and dried in a vacuum oven at 80 °C.	
RHC_f_-Mag-0.5	Ratio of FeCl_3_·6H_2_O/RHC_f_ = 0.5:1.	
RHC_f_-Mag-1	Ratio of FeCl_3_·6H_2_O/RHC_f_ = 1:1.	
RH-NCFs	RH, washed with distilled water, oven-dried at 40 °C overnight, crushed (5–10 mm), and passed through a 60-mesh screen. Soaked by a 2:1 (v/v) toluene/ethanol mixture (S:L = 30 g:450 cm^3^) for 20 h, and dried at 55 °C for 24 h. Treated with sodium chlorite solution (pH 4) at 50 °C for 1 h, and washed with distilled water. Treated with 600 cm^3^ of 5% KOH for 24 h, dried at 90 °C for 2 h, and washed with distilled water. Hydrolyzed by a mixture of (40 cm^3^ DIW + 20 cm^3^ 12.1 N HCl + 40 cm^3^ 36 N H_2_SO_4_) at 70 °C for 3 h. Sonicated at 50 KHZ at 80 °C for 3 h, and dried.	[50]
RHC-400	RH, washed and dried at 80 °C for 24 h, carbonized at 400 °C for 1.5 h, and crushed (20–80 mesh).	[51]
RHC-400-A650	RH, washed and dried at 80 °C for 24 h, carbonized at 400 °C for 0.5 h, activated by the pyrolysis technique at 650 °C for 1.5 h, and crushed (20–80 mesh).	
RH_in200–500_	RH, washed by distilled water, dried at 60 °C, ground, and sieved (200–500 µm).	[52]
RH_in500_	RH, washed by distilled water, dried at 60 °C, ground, and sieved (<500 µm).	[53]
RH_in500_	RH, washed by distilled water, dried at 60 °C, ground, and sieved (<500 µm).	[54]
RH_i_	RH, crushed, sieved, washed with distilled water, and dried at 100 °C.	[55]
RHC	RH, pyrolyzed at 350 °C for 30 min, crushed, and sieved (2 mm).	[56]
RH-X	RH, washed with distilled water and dried at 80 °C for 24 h, crushed and sieved (0.15 mm), treated with concentrated H_2_SO_4_ (S (g):L (cm^3^) = 1:3) with stirring, washed and dried overnight at room temperature, mixed with 4 M NaOH (S (g):L (cm^3^) = 1:3, contact time—90 min) with stirring, and treated with CS_2_ under optimum conditions (S (g):L (cm^3^) = 1.00, contact time—60 min, and xanthation temperature—18.5 °C).	[57]
HBC-RHs	Mix of rice husk biochar, acrylamide, N,N’-methylenebisacrylamide, and ammonium persulfate, poured into polyvinyl chloride straws (3 mm diameter), kept in an oven at 40 °C for 30 min, and left at room temperature (30 °C) for 24 h, then crushed, washed, and dried in air and then in a vacuum oven at 40 °C for 24 h.	[58]
RHIOB	RH, soaked by FeCl_3_·6H_2_O for 24 h, dried for 2 h at 80 °C, and pyrolyzed at 600 °C for 1 h under N_2_.	[59]
RH-Ti	RH, filled with a titanyl sulphate solution, heated in a water bath for 10 h, and neutralized with a potassium alkali solution or potassium liquid glass.	[60]
Powder-TiSi	RH-Ti, heated in an autoclave at ≥150 °C for 10 h, washed with distilled water, and dried at 120 °C.	
Carbon-TiSi	RH-Ti, heated separately in the tube furnace at 400, 500, 600, 700, and 800 °C for 2–3 h with water vapor blowing, washed with distilled water, and dried at 120 °C.	
ERH-CO_3_	RH, mixed with 3–5% carbonate, passed through the extruder at the exit temperature of 250–300 °C, washed with DIW, and dried at 45 °C.	[61]
Rice husk powder	RH, produced by Haitian High-Tech Material Co., Ltd., without added purification.	[62]
Expansion-treated rice husk powder	Expansion-treated rice husk powder produced by Haitian High-Tech Material Co., Ltd., without added purification.	[63]
ARH	RH, heated at 1000 °C for 3 h, cooled overnight under an Ar atmosphere, and homogenized using a three-dimensional shaker for 1 h.	[64]
ARH-250	RH, ground (size of end product = 250 μm), washed with distilled water, dried at 105 °C, heated at 1000 °C for 3 h, cooled in a desiccator, and homogenized using a three-dimensional shaker for 1 h.	[65]
CA-RH	RH, washed and dried, heated at 500 °C under an Ar atmosphere, mixed with KOH at a weight ratio of 1:5, activated at 850 °C under an Ar atmosphere, and washed with distilled water.	[66,67]
RH@MCM-41@ARS	RH, mixed with Mobil Composition of Matter No. 41 adsorbent (MCM-41) and modified by alizarin red S.	[68]
RH- H_3_PO_4_-C	RH, activated by H_3_PO_4_, and then carbonized at 700 °C for 2.5 h.	[69]
RH_wd_	RH, washed and dried without other treatment.	[70]
KHC4	RH, treated for 1 h with 25 cm^3^ of Kürschner and Hoffer reagent 4 times, filtered on a glass filter, washed with a fresh portion of Kürschner and Hoffer reagent and hot distilled water, and dried at 105 °C.	[71]
KHC4–600VA	KHC4, heated at 600 °C for 30 min, activated by water vapor at 850 °C for 30 min, treated with 70 g·dm^−3^ of NaOH at a S:L ratio of 1:10, boiled for 90 min, and washed with distilled water.	
RH_NaOH-S-gr_	RH, carbonized at 650 °C at a heating rate of 15 °C·min for 30 min, boiled with 70 g·dm^−3^ NaOH for 2 h at a S (g):L (cm^3^) ratio of 1:15, washed with distilled water, dried at 105–110 °C for 2 h, milled (0.25–0.04 mm), mixed with a 35% aqueous solution of sugar at a S (g):L (cm^3^) ratio of 1:0.35, granulated on the plate granulator for 60 min up to 0.63–2.5 mm, dried at 105–110 °C for 2 h, and carbonized at 650 °C for 30 min with concurrent activation by water vapor for 30 min at 850–900 °C.	[72]

## Data Availability

Not applicable.

## Supplementary Materials

The following supporting information can be downloaded at: https://www.mdpi.com/article/10.3390/ma16237353/s1, Table S1: Pb^2+^ ions’ removal by different rice-husk-based sorbents from mono-metal solutions; Table S2: Pb^2+^ ions’ removal by different rice-husk-based sorbents from multi-metal solutions; Table S3: Cu^2+^ ions’ removal by different rice-husk-based sorbents from multi-metal solutions; Table S4: Cd^2+^ ions’ removal by different rice-husk-based sorbents from multi-metal solutions; Table S5: Zn^2+^ ions’ removal by different rice-husk-based sorbents from mono-metal solutions; Table S6: Zn^2+^ ions’ removal by different rice-husk-based sorbents from multi-metal solutions; Table S7: Cu^2+^ ions’ removal by different rice-husk-based sorbents from mono-metal solutions; Table S8: Cd^2+^ ions’ removal by different rice-husk-based sorbents from mono-metal solutions; Table S9: Cr (VI) ions’ removal by different rice-husk-based sorbents from mono-metal solutions; Table S10: Ni^2+^ and Mn^2+^ ions’ removal by different rice-husk-based sorbents from mono-metal solutions; Table S11: Fe^2+^ ions’ removal by different rice-husk-based sorbents from mono-metal solutions; Table S12: Hg^2+^ ions’ removal by different rice-husk-based sorbents from mono- and multi-metal solutions; Table S13: Co^2+^ ions’ removal by different rice-husk-based sorbents from mono-metal solutions; Table S14: Cr^3+^, Ni^2+^, Mn^2+^, and Fe^2+^ ions’ removal by different rice-husk-based sorbents from multi-metal solutions; Table S15: As^3+^ ions’ removal by different rice-husk-based sorbents from mono-metal solutions; Table S16: Re (VII) ions’ removal by different rice-husk-based sorbents from mono-metal solutions.

## Abbreviations

The following abbreviations are used in the manuscript and Supplementary Materials:

m	Adsorbent dosage, g·dm^−3^
τ	Adsorbent–solution contact time, min
C_0_	Adsorbtive initial concentration, mg·dm^−3^
*a*	Maximum adsorption capacity, mg·g^−1^
*a_L_* (0.1_L_, 31.1_L_, etc.)	Langmuir maximum adsorption capacity, mg·g^−1^
*a_O_* (2.9_O,_ 3.5_O_, etc.)	Observed maximum adsorption capacity, mg·g^−1^
α	Removal percentage, %
*l*	Desorption efficiency after one cycle, %
IM	Isotherm models
L	Langmuir isotherm model
F	Freundlich isotherm model
R-D	Redlich–Peterson equation
l	Linear isotherm
S (g):L (cm^−3^)	Ratio of solid (g):liquid (cm^−3^)
S:L	Ratio of solid:liquid
DIW	Deionized water
v/v	Volume:volume ratio
N/D	Not detected
KM	Kinetic models
Ps1	Pseudo-first-order
Ps2	Pseudo-second-order
I-PD	Intra-particle diffusion
El	Elovich model
ΔH	Enthalpy change, kJ·mol^−1^
ΔS	Entropy change, J·mol^−1^·K^−1^
ΔG	Gibbs-free energy change, kJ·mol^−1^
