# Supplementary material for: Rice Husk-Based Adsorbents for Removal of Metals from Aqueous Solutions
Source: Materials (Basel). 2023 Nov 26;16(23):7353. doi: 10.3390/ma16237353 (PMC10706995; doi:10.3390/ma16237353)
Supplement: Supplementary file 1 [file materials-16-07353-s001.zip › materials-2705070-supplementary.pdf]

Table S1. Pb^{2+} ions removal by different rice husk-based sorbents from mono-metal solutions.

Sorbent	Conditions of experiment				*a*, mg·g^{-1}	α, %	*l*, %; eluent	IM	KM	–ΔH	–ΔS	Ref.
	C$_0$, mg·dm^{-3}	pH	m, g·dm^{-3}	τ, min								
RH$_b$	5-300	2-7	1-15	0-180	0.1$_L$	-	90; 0.2 N HNO_3	F	I-PD	–29.6 [1]	–102	[28]
RH$_{tw}$	5-200	1-5	0.1-10	0-60	31.1$_L$	-	-	L	Ps2	-	-	[29]
RH$_g$	5-500	6	1-34	0-45	21.7$_L$	-	-	L	-	14.6 [2]	–21.5 [3]	[30]
RH$_{HP}$	-	-	400	120	11.9$_L$	-	-	L	-	-	-	[31]
RH$_{TA}$	100-800	2-8	5	0-300	93.5$_L$	90	92 [4]; 0.1 M HNO_3	L	Ps2	65.5 [5]	278.8	[32]
RH$_{C\text{-}OX}$	25-200	2-6	0.5	1-80	121.8$_L$	-	90 [6]; 0.1 M HCl	L	Ps2	–4.1 [7]	–16.9	[33]
RH$_{C\text{-}Si}$ -400	25	-	4	2-120	2.9$_O$	46	-	L	Ps2	-	-	[34]
RH$_{C\text{-}Si}$-600	25	-	4	2-120	3.5$_O$	56	-	L	Ps2	-	-	
RH$_{C\text{-}Si}$-800	25	-	4	2-120	2.8$_O$	44	-	F	Ps2	-	-	
SCActA	20-100	6	10	30	0.1$_L$	-	-	F	-	-	-	[35]
RHBC	0-1036	-	5	480	29$_L$	18	-	L	-	-	-	[37]
RH$_w$	10	6	8	30	-	38	-	-	-	-	-	[38]
RH$_{wN}$	10	6	8	30	-	60	-	-	-	-	-	
RH$_{wNc}$	10	6	8	30	-	88	-	-	-	-	-	
RH$_{wNcT}$	10-100	1-10	2-20	10-50	10$_L$	99 [8] 98 [9]	97; 0.1 M HCl	L	Ps1 I-PD	32 [10]	82	
RH$_a$	50-200	2-7	3	180	58.1$_L$	-	Not necessary	L	-	-	-	[39]
RH	5-300	6.1-5.6	-	1440	20.1$_L$	-	-	-	-	-	-	[40]
RH$_{HCl}$	5-300	6.1-5.6	-	1440	18.1$_L$	-	-	-	-	-	-	
RH$_s$	5-300	6.1-5.6	-	1440	27.1$_L$	-	-	-	-	-	-	
RH$_{NaOH}$	5-300	6.1-5.6	-	1440	44.9$_L$	-	-	-	-	-	-	

[1] –ΔG = 1.5, 2.4, and 4.1 kJ·mol^{-1} at 30, 40, and 55 °C, respectively.
[2] –ΔG = 8.1, 8.7, 8.9, 9.0, 9.4, 10.0, and 10.6 kJ·mol^{-1} at 30, 35, 40, 45, 50, 55, and 60 °C, respectively.
[3] ΔS = 21.5, 19.3, 18.4, 17.6, 16.1, 14.1, and 11.2 J·mol^{-1}·K^{-1} at 30, 35, 40, 45, 50, 55, and 60 °C, respectively.
[4] 84% after 4 cycles.
[5] ΔG = 157.8, 160.6, 163.3, 166.1, and 168.9 kJ·mol^{-1} at 20, 30, 40, 50, and 60 °C, respectively.
[6] 80% after 4 cycles.
[7] –ΔG = 0.9; 1.1, and 1.3 kJ·mol^{-1} at 20, 30, and 40 °C, respectively.
[8] Artificial solution.
[9] Industrial wastewater spiked with 10 mg·dm^{-3} of Pb.
[10] Thermodynamic parameters were determined in the range of 10-50 °C. ΔG = –6.2 kJ·mol^{-1}.

Table S2. Pb^{2+} ions removal by different rice husk-based sorbents from multi-metal solutions.

Sorbent	Conditions of experiment				*a*, mg·g^{-1}	α, %	IM	KM	–ΔG	Ref.
	C$_0$, mg·dm^{-3}	pH	m, g·dm^{-3}	τ, min						
RH1	0-10 [1]	2-6	0-40	0-120	0.1$_O$	62	-	-	-	[36]
RHB	0-10 [1]	2-6	0-40	0-120	0.2$_O$	78	-	-	-	
EDTA-RHB	0-10 [1]	2-6	0-40	0-120	0.2$_O$	80	-	-	-	
MB	0-10 [1]	2-6	0-40	0-120	0.3$_O$	84	F	Ps1, Ps2	-	
RHBC	0-1036 [2]	-	5	480	16.6$_L$	11	-	-	-	[37]
RH$_{NaOH}$	40-100	-	-	1440	-	50	-	-	-	[40]
RH$_{Un}$	24.8 [3]	1.7	0.05-1.5 [4]	1-1440	-	-	L	-	–15	[41]
RHC$_a$-CO$_2$	24.8 [3]	1.7	0.05-1.5 [4]	1-1440	8$_L$	-	F	-	6.5	

[1] Metals concentrations in complex solution: Cu^{2+} - 20 mg·dm^{-3}, Cd^{2+} - 20 mg·dm^{-3}, and Pb^{2+} - 10 mg·dm^{-3}.
[2] Metals concentrations in complex solution: 5 mM for each metal involving Cu^{2+}, Cd^{2+}, Pb^{2+}, and Zn^{2+}.
[3] Metals concentrations in complex solution: Pb^{2+}, Zn^{2+}, Fe^{2+}, Mn^{2+}, and Cr^{3+} - 24.8 mg·dm^{-3}, Ni^{2+} - 50 mg·dm^{-3}.
[4] Column experiments. Column: 7 cm of height, 1 cm of diameter. Adsorbent dosage: m, g. Flow rate of 1.2 cm^3·min^{-1}.

Table S3. Cu^{2+} ions removal by different rice husk-based sorbents from multi-metal solutions.

Sorbent	Conditions of experiment				*a*, mg·g⁻¹	α, %	IM	KM	Ref.
	C_0, mg·dm⁻³	pH	m, g·dm⁻³	τ, min					
RH1	0-20 [1]	2-6	0-40	0-120	0.3_O	55	-	-	[36]
RHB	0-20 [1]	2-6	0-40	0-120	0.4_O	100	-	-	
EDTA-RHB	0-20 [1]	2-6	0-40	0-120	0.4_O	90	-	-	
MB	0-20 [1]	2-6	0-40	0-120	0.5_O	96	F, L	Ps1, Ps2, El	
RHBC	0-320 [2]	-	5	480	1.8_L	0.03	L	-	[37]
RH_{NaOH}	40-100	-	-	1440	-	50	-	-	[40]

[1] Metals concentrations in complex solution: Cu^{2+} - 20 mg·dm⁻³, Cd^{2+} - 20 mg·dm⁻³, and Pb^{2+} - 10 mg·dm⁻³.
[2] Metals concentrations in complex solution: 5 mM for each metal involving Cu^{2+}, Cd^{2+}, Pb^{2+}, and Zn^{2+}.

Table S4. Cd^{2+} ions removal by different rice husk-based sorbents from multi-metal solutions.

Sorbent	Conditions of experiment				*a*, mg·g⁻¹	α, %	IM	KM	Ref.
	C_0, mg·dm⁻³	pH	m, g·dm⁻³	τ, min					
RH1	0-20 [1]	2-6	0-40	60-120	0.3_O	52	-	-	[36]
RHB	0-20 [1]	2-6	0-40	60-120	0.4_O	82	-	-	
EDTA-RHB	0-20 [1]	2-6	0-40	60-120	0.5_O	100	-	-	
MB	0-20 [1]	2-6	0-40	60-120	0.5_O	98	F	Ps1 Ps2 El	
RHBC	0-562 [2]	-	5	480	0_L	0	-	-	[37]
RH_{NaOH}	40-100	-	-	1440	-	0	-	-	[40]

[1] Metals concentrations in complex solution: Cu^{2+} - 20 mg·dm⁻³; Cd^{2+} - 20 mg·dm⁻³; Pb^{2+} - 10 mg·dm⁻³.
[2] Metals concentrations in complex solution: 5 mM for each metal involving Cu^{2+}, Cd^{2+}, Pb^{2+}, and Zn^{2+}.

Table S5. Zn^{2+} ions removal by different rice husk-based sorbents from mono-metal solutions.

Sorbent	Conditions of experiment				*a*, mg·g⁻¹	α, %	IM	KM	Ref.
	C_0, mg·dm⁻³	pH	m, g·dm⁻³	τ, min					
RHBC	0-327	-	5	480	6.6_L	11	L	-	[37]
RH_w	10	6	8	30	-	30	-	-	[38]
RH_{wN}	10	6	8	30	-	50	-	-	
RH_{wNc}	10	6	8	30	-	80	-	-	
RH_{wNcT}	10-100	1-10	2-20	10-50	2.4_L	97 [1] N/D [2]	L	Ps1 I-PD	
RH_a	50-200	2-7	3	180	8.1_L	-	F	-	[39]
RH-500	60	3	20	0-120	1.9_O	-	-	Ps2	[42]
RHC_{AA}	0.065-1.961	-	10	-	15.3_L	-	-	-	[44]
$RH_{H2SO4wet}$	25-300	1.5-6	2	0-160	17.0_L [3]	-	L	Ps2	[45]
$RH_{H2SO4dry}$	25-300	1.5-6	2	0-160	16.0_L [4]	-	L	Ps2	

[1] Artificial solution.
[2] Industrial wastewater spiked with 10 mg·dm⁻³ of Zn.
[3] 16.978, 18.349, and 19.380 mg·g⁻¹ at 25, 35, 45 °C, respectively.
[4] 16.026, 17.889, and 18.939 mg·g⁻¹ at 25, 35, 45 °C, respectively.

Table S6. Zn^{2+} ions removal by different rice husk-based sorbents from multi-metal solutions.

Sorbent	Conditions of experiment				*a*, mg·g⁻¹	α, %	IM	−ΔG	Ref.
	C_0, mg·dm⁻³	pH	m, g·dm⁻³	τ, min					
RHBC	0-327 [1]	-	5		-	0.4	-	-	[37]
RH_{Un}	24.8 [2]	1.7	0.05-1.5 [3]	1-1440	2.3_L	-	L	11	[41]
RHC_a-CO_2	24.8 [2]	1.7	0.05-1.5 [3]	1-1440	8.2_L	-	F	7.5	

[1] Metals concentrations in complex solution: 5 mM for each metal involving Cu^{2+}, Cd^{2+}, Pb^{2+}, and Zn^{2+}.

[2] Metals concentrations in complex solution: Pb^{2+}, Zn^{2+}, Fe^{2+}, Mn^{2+}, and Cr^{3+} - 24.8 mg·dm⁻³, Ni^{2+} - 50 mg·dm⁻³.

[3] Column experiments. Column: 7 cm of height, 1 cm of diameter. Adsorbent dosage: m, g. Flow rate of 1.2 cm³·min⁻¹.

Table S7. Cu^{2+} ions removal by different rice husk-based sorbents from mono-metal solutions.

Sorbent	Conditions of experiment				*a*, mg·g⁻¹	α, %	IM	Ref.
	C_0, mg·dm⁻³	pH	m, g·dm⁻³	τ, min				
RHBC	0-320	-	5	480	4.2_L	12	L	[37]
RH_w	10	6	8	30	-	27	-	[38]
RH_{wN}	10	6	8	30	-	45	-	
RH_{wNc}	10	6	8	30	-	80	-	
RH_{wNcT}	10-100	1-10	2-20	10-50	2.3_L	93 [1]<br>N/D [2]	L	
RH_a	50-200	2-7	3	180	10.9_L		F	[39]
RH	5-300	5.55-5.73	-	1440	6.8_L	-	-	[40]
RH_{HCl}	5-300	5.55-5.73	-	1440	6.1_L	-	-	
RH_s	5-300	5.55-5.73	-	1440	6.1_L	-	-	
RH_{NaOH}	5-300	5.55-5.73	-	1440	12.5_L	-	-	
RH_{ms}	10-10000	4	10	60	41.1_L	66.7 [3]	L	[46]
ERH	0-200	1-6	10	5-120	11.4_L	86	L	[47]

[1] Artificial solution.

[2] Industrial wastewater spiked with 10 mg·cm⁻³ of Cu.

[3] *l*, %; eluent: 88.9;10% HCl.

Table S8. Cd^{2+} ions removal by different rice husk-based sorbents from mono-metal solutions.

Sorbent	Conditions of experiments				*a*, mg·g^{-1}	α, %	IM	KM	−ΔH	−ΔS	Ref.
	C_0, mg·dm^{-3}	pH	m, g·dm^{-3}	τ, min							
SCActA	20-100	6	10	90	-2.2$_{L}$	-	F	-	-	-	[35]
RHBC	0-562	-	5	480	7.8$_{L}$	10	L	-	-	-	[37]
RH$_{w}$	10	6	8	30	-	32	-	-	-	-	[38]
RH$_{wN}$	10	6	8	30	-	52	-	-	-	-	[38]
RH$_{wNc}$	10	6	8	30	-	82	-	-	-	-	[38]
RH$_{wNcT}$	10-100	1-10	2-20	10-50	5.0$_{L}$	98[1]<br>97[2]	L	Ps1<br>I-PD	29.0[3]	78.0	[38]
RH$_{a}$	50-200	2-7	3	180	16.7$_{L}$	-	F	-	-	-	[39]
RH	5-300	5.95-6.02	-	1440	7.1$_{L}$	-	-	-	-	-	[40]
RH$_{HCl}$	5-300	5.95-6.02	-	1440	3.6$_{L}$	-	-	-	-	-	[40]
RH$_{s}$	5-300	5.95-6.02	-	1440	5.0$_{L}$	-	-	-	-	-	[40]
RH$_{NaOH}$	5-300	5.95-6.02	-	1440	14.5$_{L}$	-	-	-	-	-	[40]
RH$_{i}$	20-100	2-9	0.5-3.5	10-90	21.3$_{L}$	99	F	Ps2	20.1[4]	52.0	[55]
RHC	5-200	-	5	5-120	18.7$_{L}$	-	L	Ps2	-	-	[56]
RH-X	250	7	0.5	120	138.9$_{O}$	-	-	-	-	-	[57]

[1] Artificial solution.
[2] Industrial wastewater spiked with 10 μg·cm^{-3} of Cd.
[3] Thermodynamic parameters were determined in the range of 10-50 °C. ΔG = −5.3 kJ·mol^{-1}.
[4] −ΔG = 4.7; 4.4; 3.4; 3.2 kJ·mol^{-1} at 25, 35, 45, 55 °C, respectively.

Table S9. Cr (VI) ions removal by different rice husk-based sorbents from mono-metal solutions.

Sorbent	Conditions of experiment				*a*, mg·g^{-1}	α, %	IM	KM	ΔH	ΔS	Ref.
	C_0, mg·dm^{-3}	pH	m, g·dm^{-3}	τ, min							
SCActA	20-100	6	1	30	21.6$_{O}$[1]	-	F	-	-	-	[35]
RH$_{a}$	200	2	-	0-9000	52$_{O}$[1]	-	-	-	-	-	[39]
RHP$_{450}$	10-250	3-10	0.1-1.3	0-1440	30.1$_{L}$	97	L	Ps2	-	-	[48]
RHC$_{f}$	-	-	-	-	-	-	-	-	-	-	[49]
RHC$_{f}$-Mag-2	50-300	2-11	1	0-600[2]	150.8$_{L}$[3]	-	L	Ps2	5.0[4]	19.8	[49]
RHC$_{f}$-Mag-0.5	-	-	-	-	97.0$_{L}$	-	L	-	-	-	[49]
RHC$_{f}$-Mag-1	-	-	-	-	127.0$_{L}$	-	L	-	-	-	[49]
RH-NCFs	5-50	3-10	0.2-1.5	0-120	3.8$_{L}$	93[5]	L	Ps1	100.8[6]	346.0	[50]

[1] Cr (III).
[2] To determine some adsorption parameters, experiments were carried out for 1440 min.
[3] 150.8, 153.4, and 157.7 mg·g^{-1} at 25, 35, 45 °C, respectively.
[4] −ΔG = 0.9; 1.1; 1.3 kJ·mol^{-1} at 25, 35, and 45 °C, respectively.
[5] *l*, %; eluent: 80 and 50 after 1 and 4 cycles, respectively; 0.5 M HNO_3.
[6] −ΔG = 0.2; 0.2; 0.2 kJ·mol^{-1} at 10, 20, and 30 °C, respectively.

Table S10. Ni^{2+} and Mn^{2+} ions removal by different rice husk-based sorbents from mono-metal solutions.

Sorbent	Metal	Conditions of experiments				*a*, mg·g⁻¹	IM	Ref.
		C_0, mg·dm⁻³	pH	m, g·dm⁻³	τ, min			
RH_a	Ni^{2+}	50-200	2-7	3	180	5.5_L	L	[39]
	Mn^{2+}	50-200	2-7	3	180	8.3_L	L	

Table S11. Fe^{2+} ions removal by different rice husk-based sorbents from mono-metal solutions.

Sorbent	Conditions of experiment				*a*, mg·g⁻¹	α, %	Ref.
	C_0, mg·dm⁻³	pH	m, g	τ, min			
RH	5-300	3-4.35	-	1440	4.7_L [1]	-	[40]
RH_{HCl}	5-300	3-4.35	-	1440	1.7_L [1]	-	
RH_s	5-300	3-4.35	-	1440	19.0_L [1]	-	
RH_{NaOH}	5-300	3-4.35	-	1440	7.6_L [1]	-	
RHC-400	3	-	50 [2]	20 [2]	-	55	[51]
				40 [2]	-	55	
				60 [2]	-	64	
				80 [2]	-	64	
				100 [2]	-	64	
				120 [2]	-	65	
				180 [2]	-	66	
RHC-400-A650	3	-	50 [2]	20 [2]	-	64	[51]
				40 [2]	-	65	
				60 [2]	-	72	
				80 [2]	-	69	
				100 [2]	-	71	
				120 [2]	-	70	
				180 [2]	-	74	

[1] Fe (III).

[2] Column experiments. Column: 40 cm of height, 5.4 cm of diameter. Flow rate of 1 dm³·min⁻¹.

Table S12. Hg^{2+} ions removal by different rice husk-based sorbents from mono- and multi-metal solutions.

Sorbent	Conditions of experiment				*a*,	α,	IM	KM	Ref.
	C_0, mg·dm^{-3}	pH	m, g·dm^{-3}	τ, min	mg·g^{-1}	%			
RH_a	50-200	2-7	3	180	36.1_L	-	L	-	[39]
$RH_{H2SO4wet}$	100-1500	1.5-6	1.5	0-120	303.0_L [1]	-	L	Ps2	[45]
$RH_{H2SO4dry}$	100-1500	1.5-6	1.5	0-120	227.3_L [2]	-	L	Ps2	
$RH_{in200\text{-}500}$	0.05, 0.5	6-7	0.5	0-10200	-	93 [3]	-	-	[52]
RH_{in500}	0.05, 0.5	5.7-6	0.005-1.0	0-10200	$0.5\text{-}24.1_O$ [4]	24-94 [4]	l	El	[53]
RH_{in500}	0.05, 0.5	3, 6, 9	0.5	0-10200	38.1_O [5]	91.9 [5]	-	-	[54]

[1] 303.0, 336.7, and 384.6 mg·g^{-1} at 25, 35, 45 °C, respectively.
[2] 227.3, 270.3, and 303.0 mg·g^{-1} at 25, 35, 45 °C, respectively.
[3] Data for river water spiked with 0.5 mg·dm^{-3} Hg (II) are shown.
[4] Data for C_0 (Hg^{2+}) = 0.5 g·dm^{-3} are shown. The minimum and maximum *a* and α values correspond to 0.005 and 1.0 g·dm^{-3} of adsorbent dosage, respectively.
[5] Data for C_0 (Hg^{2+}) = 0.5 g·dm^{-3} are shown. There were *a* = 39.1, 46.3, and 20.0 mg·g^{-1} and α = 92, 90, and 96% in the presence of 0.1 M $NaNO_3$, 0.5 M $NaNO_3$, and 0.5 M NaCl, respectively. There were *a* = 25.2 and 36.2 mg·g^{-1} and α = 95 and 92 % when C_0 (Hg): C_0 (Cd) ratio was 1:4 and 1:1, respectively. There were *a* = 62.1 and 47.6 mg·g^{-1} and α = 81 and 83% in the presence of 5 and 10 mg·dm^{-3} humic substances, respectively. There were *a* = 33.7 mg·g^{-1} and α = 85% in river water spiked with 0.5 mg·dm^{-3} Hg (II).

Table S13. Co^{2+} ions removal by different rice husk-based sorbents from mono-metal solutions.

Sorbent	Conditions of experiment				*a*,	α,	*l*, %;	IM	Ref.
	C_0, mg·dm^{-3}	pH	m, g·dm^{-3}	τ, min	mg·g^{-1}	%	eluent		
RH_a	50-200	2-7	3	180	9.6_L	-	Not necessary	L	[39]
RH_{ms}	10-10000	4	10	60	494.2_L	6-73 [1]	28<br>10% HCl	L	[46]

[1] There were α = 6% at C_0 = 10000 mg·dm^{-3} and α = 73% at C_0 = 10 mg·dm^{-3}.

Table S14. Cr^{3+}, Ni^{2+}, Mn^{2+}, and Fe^{2+} ions removal by different rice husk-based sorbents from multi-metal solutions.

Sorbent	Metal	Conditions of experiment				*a*,	IM	−ΔG	Ref.
		C_0, mg·dm^{-3}	pH	m, g	τ, min	mg·g^{-1}			
RH_{Un}	Cr^{3+}	24.8 [1]	1.7	0.05-1.5 [2]	1-1440	40.2_L	F	9	[41]
	Ni^{2+}	50 [1]				1.7_L	L	9	
	Mn^{2+}	24.8 [1]				216_L	F	0	
	Fe^{2+}	24.8 [1]				206_L	F	4.5	
$RHC_a\text{-}CO_2$	Cr^{3+}	24.8 [1]				37.2_L	F	9	
	Ni^{2+}	50 [1]				9.6_L	F	8.8	
	Mn^{2+}	24.8 [1]				23.9_L	F	6	
	Fe^{2+}	24.8 [1]				29.6_L	F	8.8	

[1] Metals concentrations in complex solution: Pb^{2+}, Zn^{2+}, Fe^{2+}, Mn^{2+}, and Cr^{3+} - 24.8 mg·dm^{-3}, Ni^{2+} - 50 mg·dm^{-3}.
[2] Column experiments. Column: 7 cm of height, 1 cm of diameter. Flow rate of 1.2 cm^3·min^{-1}.

Table S15. As^{3+} ions removal by different rice husk-based sorbents from mono-metal solutions.

Sorbent	Conditions of experiment				*a*, mg·g⁻¹	α, %	*l*, %; eluent	IM	KM	Ref.
	C_0, mg·dm^{-3}	pH	m, g·dm^{-3}	τ, h						
HBC-RHs	1-150	4-10	0.167-16.67	0.5-48	28.3_L	~100	-	L	Ps2	[58]
RHIOB	0.05-0.2	2-10	0.5-2	0.5-48	0.1_L [1]	90	90 [2]; 0.1 N NaON+ 0.1 N NaCl	R-P	Ps2	[59]

[1] Studies were carried out at high arsenic concentrations.

[2] 86% after 4 cycles.

Table S16. Re (VII) ions removal by different rice husk-based sorbents from mono- and multi-metal solutions.

Sorbent	Conditions of experiment				*a*, mg·g^{-1}	α, %	*l*, %; eluent	IM	Ref.
	C_0, mg·dm^{-3}	pH	m, g·dm^{-3}	τ, min					
SCActA	20-100	6	10	30	8.9_L	-	-	L	[35]
KHC4	-	-	-	-	-	-	-	-	[71]
KHC4-600VA	20-100	6	10	30	4.0_L [1]	90	-	F	
$RH_{NaOH\text{-}S\text{-}gr}$	35 [2]	-	2	60-480	13_O; 23.4_O [3]	75	70; 8%NH_4OH	-	[74]

[1] a_O = 8.8 mg·dm^{-3} in the 60 min process.

[2] Industrial multi-metal solution containing 35 mg·dm^{-3} of Re (VII).

[3] Column experiments. Column: 1 cm of diameter. Adsorbent dosage: 2, g. Adsorbent volume: 9.5 cm^3. Flow rates of 100 cm^3·h^{-1}.
